# Burden of migraine in Finland: multimorbidity and phenotypic disease networks in occupational healthcare

**DOI:** 10.1186/s10194-020-1077-x

**Published:** 2020-01-31

**Authors:** Minna A. Korolainen, Samuli Tuominen, Samu Kurki, Mariann I. Lassenius, Iiro Toppila, Timo Purmonen, Jaana Santaholma, Markku Nissilä

**Affiliations:** 1 Novartis Finland Oy, Espoo, Finland; 2 Medaffcon Oy, Espoo, Finland; 3 Terveystalo Biobank Finland, Turku, Finland

**Keywords:** Diseasome, Multimorbidity, Migraine, Networks, ICD-10, Occupational healthcare

## Abstract

**Background:**

Migraine is a complex neurological disorder with high co-existing morbidity burden. The aim of our study was to examine the overall morbidity and phenotypic diseasome for migraine among people of working age using real world data collected as a part of routine clinical practice.

**Methods:**

Electronic medical records (EMR) of patients with migraine (*n* = 17,623) and age- and gender matched controls (*n* = 17,623) were included in this retrospective analysis. EMRs were assessed for the prevalence of ICD-10 codes, those with at least two significant phi correlations, and a prevalence >2.5% in migraine patients were included to phenotypic disease networks (PDN) for further analysis. An automatic subnetwork detection algorithm was applied in order to cluster the diagnoses within the PDNs. The diagnosis-wise connectivity based on the PDNs was compared between migraine patients and controls to assess differences in morbidity patterns.

**Results:**

The mean number of diagnoses per patient was increased 1.7-fold in migraine compared to controls. Altogether 1337 different ICD-10 codes were detected in EMRs of migraine patients. Monodiagnosis was present in 1% and 13%, and the median number of diagnoses was 12 and 6 in migraine patients and controls. The number of significant phi-correlations was 2.3-fold increased, and cluster analysis showed more clusters in those with migraine vs. controls (9 vs. 6). For migraine, the PDN was larger and denser and exhibited one large cluster containing fatigue, respiratory, sympathetic nervous system, gastrointestinal, infection, mental and mood disorder diagnoses. Migraine patients were more likely affected by multiple conditions compared to controls, even if no notable differences in morbidity patterns were identified through connectivity measures. Frequencies of ICD-10 codes on a three character and block level were increased across the whole diagnostic spectrum in migraine.

**Conclusions:**

Migraine was associated with an increased multimorbidity, evidenced by multiple different approaches in the study. A systematic increase in the morbidity across the whole spectrum of ICD-10 coded diagnoses, and when interpreting PDNs, were detected in migraine patients. However, no specific diagnoses explained the morbidity. The results reflect clinical praxis, but also undoubtedly, the pathophysiological phenotypes related to migraine, and emphasize the importance of better understanding migraine-related morbidity.

## Introduction

Multimorbidity, defined as the co-occurrence of two or more diseases or conditions in an individual, has been described in migraine [1–4]. Although multimorbidity generally increases with age, comorbidities are present already in pediatric migraine [5]. The importance of multimorbidity is emphasized by an increase in health and social care expenditure per additional morbidity [6]. Global burden of disease repeatedly identifies migraine as one of the top conditions resulting in years lived with disability, likely attributable to the multimorbid strain on individuals [7, 8].

Physical and mental comorbidity in migraine has been examined extensively, and yet remain intricate. The evidence is mostly based on pairwise comparisons or examination of targeted conditions. A large number of migraine-associated comorbidities have been identified including asthma and allergies, psychiatric, cardiovascular, cerebrovascular, gastrointestinal, musculoskeletal disorders, as well as other neurological and pain-related disorders [9–19]. Moreover, several symptoms related to migraine attacks such as sensitivity to light, sound and odours also occur to some extent between migraine attacks. This may be explained by a lower general discomfort threshold in response to sensory stimuli, leading to enhanced perception of both painful and non-painful sensations [20]. Migraine is acknowledged as a complex genetic disorder that runs in families. Current evidence from genome-wide association studies mainly point at vascular function and metal ion channel activity involvement in the pathophysiology, whereas less genes linking to neuronal function and ion channel activity have been found [21].

The prevalence of multimorbidity has been estimated to be 13–83%, and multimorbidity, as such, is understudied and underpublished [22]. Networks underlying multimorbidity are complex. Sturmberg and colleagues argued multimorbidity being “the manifestation of interconnected physiological network processes within an individual in his or her socio-cultural environment” [23]. This very well describes the complexity in understanding holistic and personalised disease patterns in individuals when these networks include multiple -omics, neuroendocrine, immune cell and mitochondrial energy-related elements as well as social, environmental, and healthcare networks.

Human diseasome is thought to comprehend disease genome and disease phenome. Phenotypic disease networks (PDN) provide a holistic view over a condition and the related multimorbidity. Previously PDNs have been used to study the multimorbidity patterns underlying depression as well as heart failure, migraine, diabetes and dementia in elderly patients [3, 24]. There migraine has been shown to be comorbid with for example depression, diabetes mellitus, and irritable bowel syndrome. Disease progression has been studied by assessing the directionality of PDNs as well as the association between the lethality and the connectivity in a PDN of a given condition [25]. The results suggested that patients are more likely to be affected by conditions that are close to the conditions already affecting them in a PDN, however, migraine was not present in the analyses.

We have previously shown that migraine was associated with a 1.7-fold increase in healthcare resource utilization and 1.8-fold increase sick-leave days when compared to age and gender matched controls. Less than 10% of visits and sick-leaves were linked directly to migraine, and we detected that some of the diagnoses such as depression and anxiety were more frequent among migraine patients when compared to age- and gender-matched controls. Notably, the disease burden including the frequency of some additional co-existing morbidities increased by failing prophylactic treatments [26].

The aim of the current study was to further investigate comprehensive patterns of morbidity based on ICD-10 coded phenotypic diseasomes in migraine patients compared to age- and gender-matched controls. Migraine was associated with significant increase in overall morbidity seen both as increased multimorbidity across the ICD-10 coded diagnostic spectrum and in the larger PDN, in which diagnoses clustered differentially between migraine patients and controls. These findings strongly point at a significant multimorbidity among migraine patients that may reflect the polygenic nature of migraine but also complex representation of migraine symptoms in ICD-10 coded clinical praxis.

## Material and methods

This retrospective register study included migraine patients using occupational healthcare with a G43* diagnosis in the electronic medical records (EMR) between 1st January 2012 and 31st December 2017 at the private healthcare provider Terveystalo. Altogether, 17,623 of the patients had migraine according to ICD-10 code (G43*, on a three-character level) and were included to this study. A one-to-one, age- and gender-matched control population without migraine was created. For each migraine patient a control patient was randomly chosen based on gender and birth date from the database. No subjects were chosen twice for the control group [Controls: *N* = 17,623, 76,804 patient-years, average age 38.9 years, 78.9% female; migraine patients: *N* = 17,623, 51,396 patient-years, average age 38.9 years, 78.9% female]. For both migraine patients and controls, diagnoses were assessed from the EMR during the study period, irrespective of their timing in relation to the migraine diagnosis. Controls were used as reference for comorbidity estimations. The study cohort has been described in detail previously [26].

Disease networks and clustering were done at an ICD-10 code three-character level e.g. H01*. Diagnostic codes with a prevalence of 2.5–20% in migraineurs were included in the network analysis. The most common diagnostic codes (prevalence > 20% in the migraine patients) were excluded from the network analyses but assessed separately, as these high prevalence morbidities would have been the main drivers for cluster formations, which would have resulted in yielding worse clusters as assessed by modularity.

Phi-correlations were calculated between 205 and 105 diagnostic codes in migraine patients and controls, respectively. The phi-correlations and the statistical significance were calculated following Hidalgo et al. 2009 [25]. Phi correlation was chosen over the relative risk ratio due to more convenient numeric properties i.e. approximate normal distribution without requiring any transformations. Briefly, phi correlation is calculated like the regular Pearson correlation, but between two binary variables, here if a patient was or was not recorded with a given diagnosis code. Thus, the possible values of the phi correlation range from − 1 to 1. Phi correlation − 1 between two diagnosis codes mean that exactly the patients that were recorded with the diagnosis code 1 were not recorded with the diagnosis code 2, and conversely for the diagnosis code 2. Phi correlation 1 means that exactly the same patients were recorded with both diagnosis code 1 and 2. Phi correlation 0 means that there was no correlation between the diagnosis codes. The significance level 0.05 was used throughout and the significance was calculated as in Hidalgo et al. 2009 [25]. Furthermore, co-existing morbidities that significantly correlated with only one other morbidity were excluded from clustering and the network visualizations.

An automatic cluster detection method called the Walktrap-algorithm was applied in order to distinguish more closely related subsets of potential comorbidities [27]. The Walktrap-algorithm uses short random walks within the network weighted by the phi-correlations. One random walk consists of first selecting a diagnosis code at random and then again randomly selecting another diagnosis code that has a phi correlation with the current diagnosis code. Diagnosis codes with higher phi correlations are more likely to be selected as the second diagnosis code. The second selected diagnosis code becomes the current diagnosis code completing one step. These steps were repeated a specified number of times which is called a random walk. Diagnosis codes that are often part of the same random walk clustered together. Random walk lengths of 4 were used in this study.

The network visualizations utilize a spring layout where comorbidities with higher phi-correlations are placed closer to each other. The modularity and four diagnosis code - wise centrality measures, namely the degree, betweenness, closeness and strength, were calculated, reported, and visualized [28]. Explanations of the calculation and interpretation of the centrality measures are included in Appendix 1 [27, 28]. The degree distributions of migraineurs and controls were compared using a scatterplot and by regressing the number of significant phi-correlations in migraineurs on the controls. Outliers were detected using the mean-shift test at significance level 0.05 with Bonferroni correction.

The number of distinct diagnoses per person was assessed from ICD-10 codes for controls and patients with migraine. The frequency of patients per number of diagnosis codes for diagnosis codes included in the PDNs were reported. Overall diagnoses were further assessed at a block level (e.g. H53-H54, visual disturbances and blindness) in migraine patients vs. controls. Blocks with prevalence over 2% in migraine patients and fold change of at least 1.5 were reported. The prevalence differences between migraine patients and controls were tested with Chi-squared test at significance level 0.05. Baseline characteristics are presented at the time of the first G43* diagnosis.

All analyses were conducted using R: A language and environment for statistical computing, version 3.5.2. The network analyses and visualizations used the qgraph and igraph packages available on the Comprehensive R Archive Network (CRAN).

## Results

Patients with migraine were on average 39 years, and the majority were women (Table 1). Prophylactic migraine medication was prescribed to 13% of the cohort, acute migraine medication to 37% and 51% had no migraine prescriptions from the occupational healthcare [26]. Controls were lacking G43*diagnosis, and matched based on age and gender. The median follow-up time was 2.5 years longer for controls than for migraine patients. The study provides new insight on increased multimorbidity across all diagnosis codes in migraine and shows that diagnoses cluster differentially between migraine patients and controls in phenotypic disease networks.

**Table 1:** Baseline characteristics of the migraine patients and controls

	Controls	Migraine (G43*)
Follow-up time, median (min-max)	5.19 (0.11–6.00)	2.84 (0.09–6.00)
N	17,623	17,623
Female, %	78.9%	78.9%
Age, mean	39 (15–77)	39 (15–77)

**Fig. 1:**
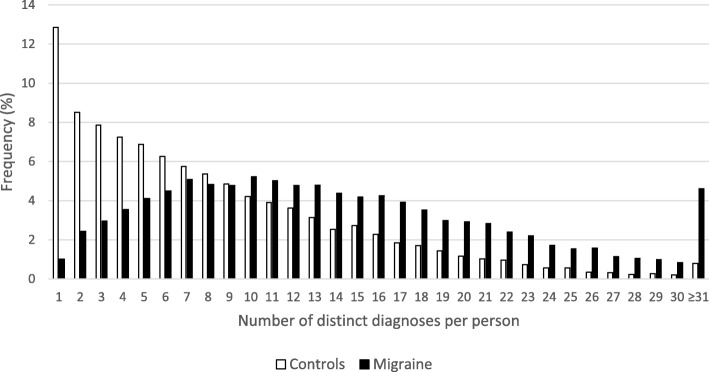
The frequency of patients per distinct number of diagnoses per patient among patients with migraine and controls

Altogether 1337 different ICD-10 codes were detected in EMRs, but all were not included in the further analyses due to low abundancy. The mean number of diagnoses per patient was increased 1.7-fold in migraine compared to controls. The median number of distinct diagnoses per person was 12 for migraine patients and 6 for controls. In the migraine patients 1.0% had only one diagnosis (i.e. monomorbidity) whereas 12.8% of controls had only one diagnosis. A histogram with the frequency of patients or controls per number of distinct diagnoses per person is presented in Fig. 1.

**Fig. 2:**
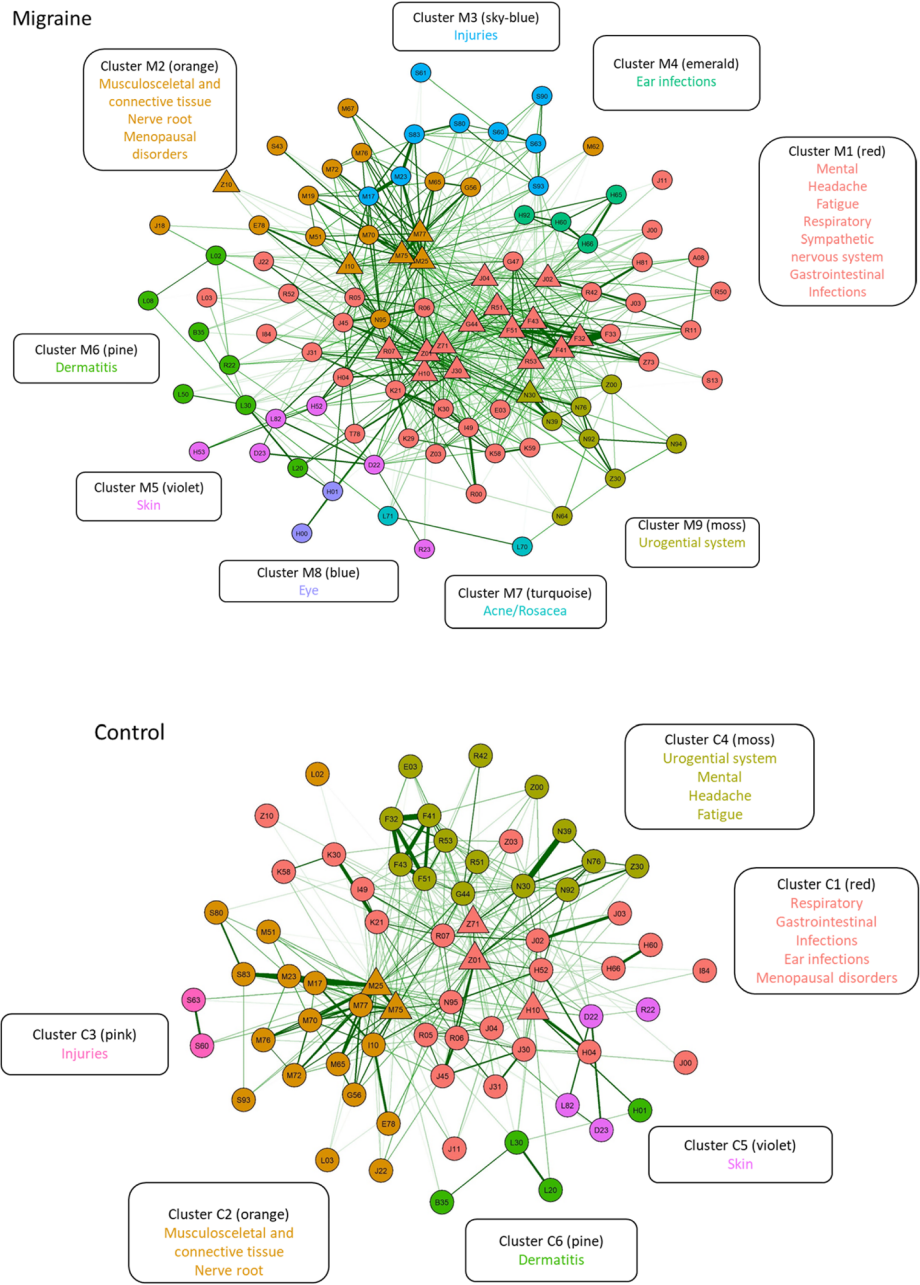
Comorbidity networks in patients with migraine (upper panel) and age and gender matched controls (lower panel). The visualizations utilize the spring layout where diagnoses with higher phi-correlation are placed closer to each other. Note that only phi-correlations above 0.04 have been visualized to avoid cluttering, however, all statistically significant phi-correlations have been utilized in placing the comorbidities

The individual diagnoses that are present in comorbidity networks are shown in in Table 2 and the PDNs in Fig. 2. Moreover, the frequencies for each of these diagnoses exhibited a significant increase among migraine patients when compared to controls (Table 2). The number of significant phi-correlations (*p* < 0.05) was greater in patients with migraine than among controls (4752 vs. 2804). There were 197 potential co-existing morbidities in migraine patients and 148 morbidities in controls with at least two significant phi-correlations. The median number of significant phi-correlations per diagnosis code was 12 and 9 for migraine patients and controls, respectively.

**Table 2:** List of diagnosis clusters from Fig. 2 sorted by their presence in migraine patients (2.5–20% prevalence) and compared to controls. Fold change column shows the prevalence in migraineurs divided by the prevalence in controls. All fold changes are greater than 1 indicating that migraine is associated with higher multimorbidity compared to controls. Due to high sample size, the difference in prevalence between migraineurs and controls is statistically significant (*p* < 0.001) for each diagnosis

Code	Migraine cluster	Control cluster	Name	M (%)	C (%)	Fold change
A08	M1	N/A	Viral and other specified intestinal infections	4.10	1.80	2.28
E03	M1	C4	Other hypothyroidism	5.20	3.30	1.58
F32	M1	C4	Depressive episode	13.00	7.10	1.83
F33	M1	N/A	Recurrent depressive disorder	4.80	2.50	1.92
F41	M1	C4	Other anxiety disorders	14.10	7.90	1.78
F43	M1	C4	Reaction to severe stress, and adjustment disorders	17.40	9.90	1.76
F51	M1	C4	Nonorganic sleep disorders	17.40	8.80	1.98
G44	M1	C4	Other headache syndromes	19.50	5.90	3.31
G47	M1	N/A	Sleep disorders	4.60	2.40	1.92
H04	M1	C1	Disorders of lacrimal system	4.10	2.50	1.64
H10	M1	C1	Conjunctivitis	16.20	11.00	1.47
H81	M1	N/A	Disorders of vestibular function	4.50	2.30	1.96
I49	M1	C1	Other cardiac arrhythmias	6.60	3.90	1.69
I84	M1	C1	Haemorrhoids	5.30	3.20	1.66
J00	M1	C1	Acute nasopharyngitis [common cold]	6.00	3.60	1.67
J02	M1	C1	Acute pharyngitis	15.50	9.40	1.65
J03	M1	C1	Acute tonsillitis	9.90	6.50	1.52
J04	M1	C1	Acute laryngitis and tracheitis	10.20	5.90	1.73
J11	M1	C1	Influenza, virus not identified	6.10	3.70	1.65
J22	M1	C2	Unspecified acute lower respiratory infection	4.50	2.60	1.73
J30	M1	C1	Vasomotor and allergic rhinitis	10.80	6.50	1.66
J31	M1	C1	Chronic rhinitis, nasopharyngitis and pharyngitis	5.00	2.90	1.72
J45	M1	C1	Asthma	8.40	5.40	1.56
K21	M1	C1	Gastro-oesophageal reflux disease	7.10	4.00	1.78
K29	M1	N/A	Gastritis and duodenitis	3.60	1.80	2.00
K30	M1	C1	Dyspepsia	6.00	3.00	2.00
K58	M1	C1	Irritable bowel syndrome	4.80	2.50	1.92
K59	M1	N/A	Other functional intestinal disorders	3.60	1.90	1.89
L03	M1	C2	Cellulitis	4.60	2.70	1.70
R00	M1	N/A	Abnormalities of heart beat	3.30	1.80	1.83
R05	M1	C1	Cough	8.90	5.90	1.51
R06	M1	C1	Abnormalities of breathing	6.30	3.80	1.66
R07	M1	C1	Pain in throat and chest	11.20	6.50	1.72
R11	M1	N/A	Nausea and vomiting	3.90	1.60	2.44
R42	M1	C4	Dizziness and giddiness	7.10	3.40	2.09
R50	M1	N/A	Fever of other and unknown origin	4.90	2.70	1.81
R51	M1	C4	Headache	18.10	4.10	4.41
R52	M1	N/A	Pain, not elsewhere classified	3.20	1.70	1.88
R53	M1	C4	Malaise and fatigue	12.20	6.70	1.82
S13	M1	N/A	Dislocation, sprain and strain of joints and ligaments at neck level	2.60	1.30	2.00
T78	M1	N/A	Adverse effects, not elsewhere classified	3.20	1.80	1.78
Z01	M1	C1	Other special examinations and investigations of persons without complaint or reported diagnosis	19.80	14.10	1.40
Z03	M1	C1	Medical observation and evaluation for suspected diseases and conditions	6.20	3.60	1.72
Z71	M1	C1	Persons encountering health services for other counselling and medical advice, not elsewhere classified	15.60	10.00	1.56
Z73	M1	N/A	Problems related to life-management difficulty	3.20	1.50	2.13
E78	M2	C2	Disorders of lipoprotein metabolism and other lipidaemias	4.40	3.20	1.38
G56	M2	C2	Mononeuropathies of upper limb	4.50	2.90	1.55
I10	M2	C2	Essential (primary) hypertension	11.30	8.70	1.30
J18	M2	N/A	Pneumonia, organism unspecified	3.20	2.50	1.28
M19	M2	N/A	Other arthrosis	2.50	1.80	1.39
M25	M2	C2	Other joint disorders	19.60	12.80	1.53
M51	M2	C2	Other intervertebral disc disorders	6.70	4.10	1.63
M62	M2	N/A	Other disorders of muscle	3.50	1.80	1.94
M65	M2	C2	Synovitis and tenosynovitis	7.00	4.60	1.52
M67	M2	N/A	Other disorders of synovium and tendon	3.20	2.10	1.52
M70	M2	C2	Soft tissue disorders related to use, overuse and pressure	8.00	4.90	1.63
M72	M2	C2	Fibroblastic disorders	5.40	3.60	1.50
M75	M2	C2	Shoulder lesions	19.90	13.30	1.50
M76	M2	C2	Enthesopathies of lower limb, excluding foot	4.70	3.10	1.52
M77	M2	C2	Other enthesopathies	15.30	9.90	1.55
N95	M2	C1	Menopausal and other perimenopausal disorders	7.90	5.20	1.52
S43	M2	N/A	Dislocation, sprain and strain of joints and ligaments of shoulder girdle	3.40	2.10	1.62
Z10	M2	C1	Routine general health check-up of defined subpopulation	10.60	8.90	1.19
M17	M3	C2	Gonarthrosis [arthrosis of knee]	4.50	3.50	1.29
M23	M3	C2	Internal derangement of knee	7.10	4.80	1.48
S60	M3	C3	Superficial injury of wrist and hand	5.40	3.10	1.74
S61	M3	N/A	Open wound of wrist and hand	4.00	2.90	1.38
S63	M3	C3	Dislocation, sprain and strain of joints and ligaments at wrist and hand level	5.20	3.40	1.53
S80	M3	C2	Superficial injury of lower leg	4.50	3.00	1.50
S83	M3	C2	Dislocation, sprain and strain of joints and ligaments of knee	6.20	4.50	1.38
S90	M3	N/A	Superficial injury of ankle and foot	4.00	2.30	1.74
S93	M3	C2	Dislocation, sprain and strain of joints and ligaments at ankle and foot level	8.60	5.60	1.54
H60	M4	C1	Otitis externa	6.90	4.50	1.53
H65	M4	N/A	Nonsuppurative otitis media	3.40	2.10	1.62
H66	M4	C1	Suppurative and unspecified otitis media	8.80	5.90	1.49
H92	M4	N/A	Otalgia and effusion of ear	3.10	1.80	1.72
D22	M5	C5	Melanocytic naevi	9.90	7.10	1.39
D23	M5	C5	Other benign neoplasms of skin	4.40	3.20	1.38
H52	M5	C1	Disorders of refraction and accommodation	6.90	4.00	1.73
H53	M5	N/A	Visual disturbances	4.20	1.40	3.00
L82	M5	C5	Seborrhoeic keratosis	3.90	2.60	1.50
R23	M5	N/A	Other skin changes	2.80	1.70	1.65
B35	M6	C6	Dermatophytosis	4.20	3.20	1.31
L02	M6	C2	Cutaneous abscess, furuncle and carbuncle	4.80	3.10	1.55
L08	M6	N/A	Other local infections of skin and subcutaneous tissue	3.00	2.00	1.50
L20	M6	C6	Atopic dermatitis	5.80	4.20	1.38
L30	M6	C6	Other dermatitis	8.90	6.50	1.37
L50	M6	N/A	Urticaria	3.50	2.30	1.52
R22	M6	C5	Localized swelling, mass and lump of skin and subcutaneous tissue	5.90	4.00	1.48
L70	M7	N/A	Acne	4.10	2.40	1.71
L71	M7	N/A	Rosacea	3.00	1.60	1.88
H00	M8	N/A	Hordeolum and chalazion	3.30	2.00	1.65
H01	M8	C6	Other inflammation of eyelid	4.10	2.50	1.64
N30	M9	C4	Cystitis	13.50	9.00	1.50
N39	M9	C4	Other diseases of urinary system	6.70	4.40	1.52
N64	M9	N/A	Other disorders of breast	2.50	1.60	1.56
N76	M9	C4	Other inflammation of vagina and vulva	5.70	3.90	1.46
N92	M9	C4	Excessive, frequent and irregular menstruation	9.20	6.90	1.33
N94	M9	N/A	Pain and other conditions associated with female genital organs and menstrual cycle	3.10	1.60	1.94
Z00	M9	C4	General examination and investigation of persons without complaint and reported diagnosis	8.60	5.30	1.62
Z30	M9	C4	Contraceptive management	6.40	5.10	1.25

Altogether nine clusters were formed for migraine patients (Fig. 2). A large cluster M1 shown in Fig. 2, emerged where the diagnoses that were linked with each other included mental disorders (e.g. anxiety F41, fatigue R53, stress-related disorder F43) respiratory disorders (e.g. asthma J45, cough R05), sympathetic nervous system disorders (e.g. disorders of the vestibular system H81, disorders of the lacrimal system H04), infections (e.g. J03 acute tonsillitis, R50 fever), and gastrointestinal problems (e.g. IBS K58, dyspepsia K30, gastritis and duodenitis K29). Another cluster M2 was formed of musculoskeletal and connective tissue, nerve root and menopausal diagnoses. The other seven clusters consisted of more homogeneous diagnostic codes that could be explained by single nominators including injuries (M3), ear infections (M4), dermatitis (M6), acne/rosacea (M7), eye (M8), skin (M5), and urogenital disorders (M9).

The comorbidity network was smaller in controls consisting of six clusters (vs. nine in migraine). Further differences included clusters C1, C2, and C4 that consisted of more heterogeneous diagnosis codes as well as clusters with diagnostic codes that could be identified by single nominators such as injuries (C3), dermatitis (C6) and skin disorders (C5) (Fig. 2 and Table 2).

**Fig. 3:**
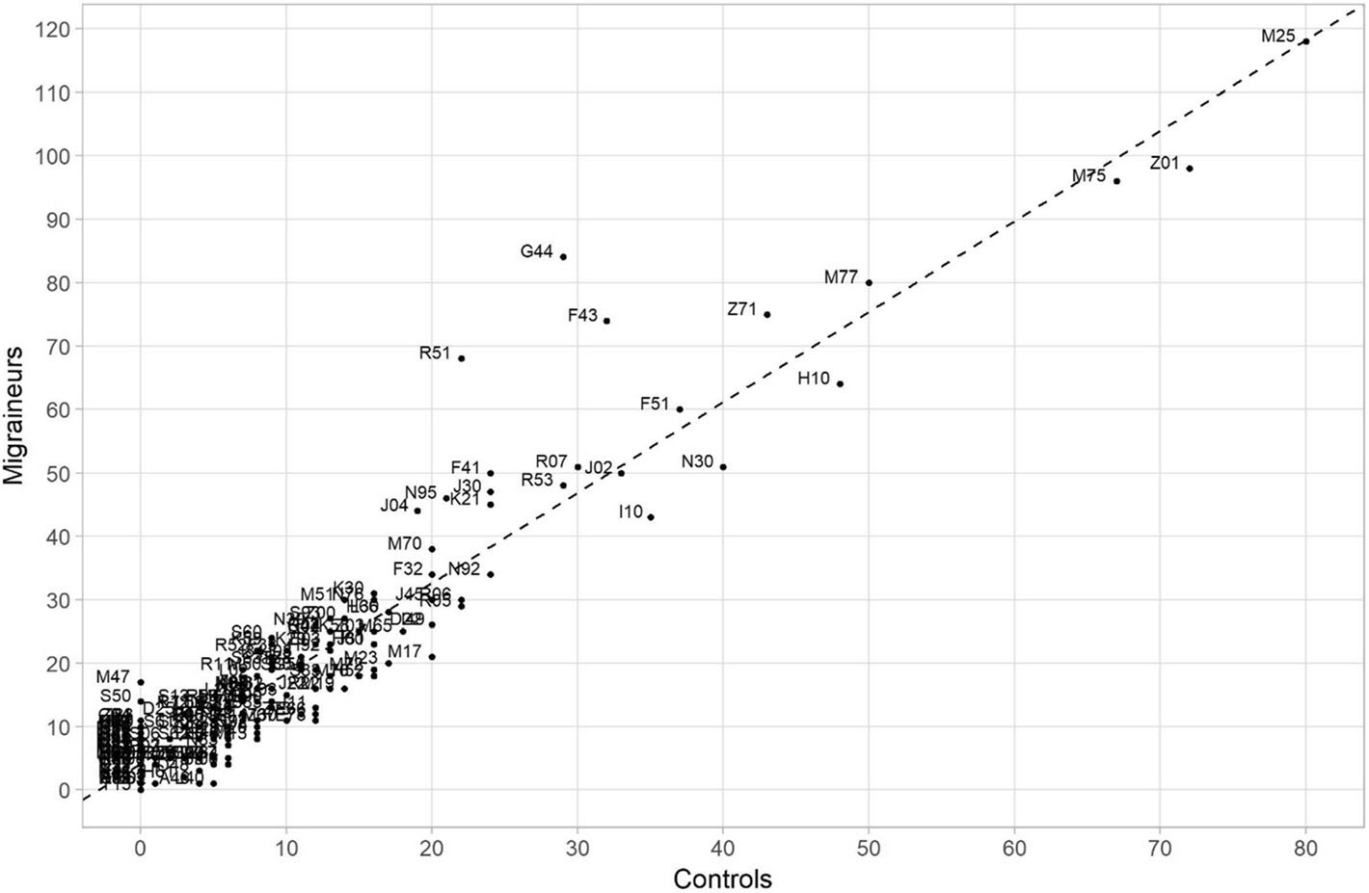
Number of statistically significant phi-correlations per each comorbidity (degree) plotted in migraineurs vs controls. The dashed line represents the regression slope from regressing the number of significant phi-correlations in migraineurs on the controls. Slope = 1.43 (*p* < 0.001), intercept = 4.04 (*p* < 0.001), R-squared = 88.6%. The fit shows that the number of significant phi-correlations per comorbidity in migraineurs is explained quite well by the respective number in controls, when the overall increase in morbidity in migraineurs is accounted for. However, visual assessment suggests 3 outliers: F43 (reaction to severe stress, and adjustment disorders), G44 (other headache syndromes), and R51 (headache). This is confirmed by a formal mean-shift outlier test (Bonferroni corrected *p*-values < 0.001 for all three diagnosis codes)

**Fig. 4:**
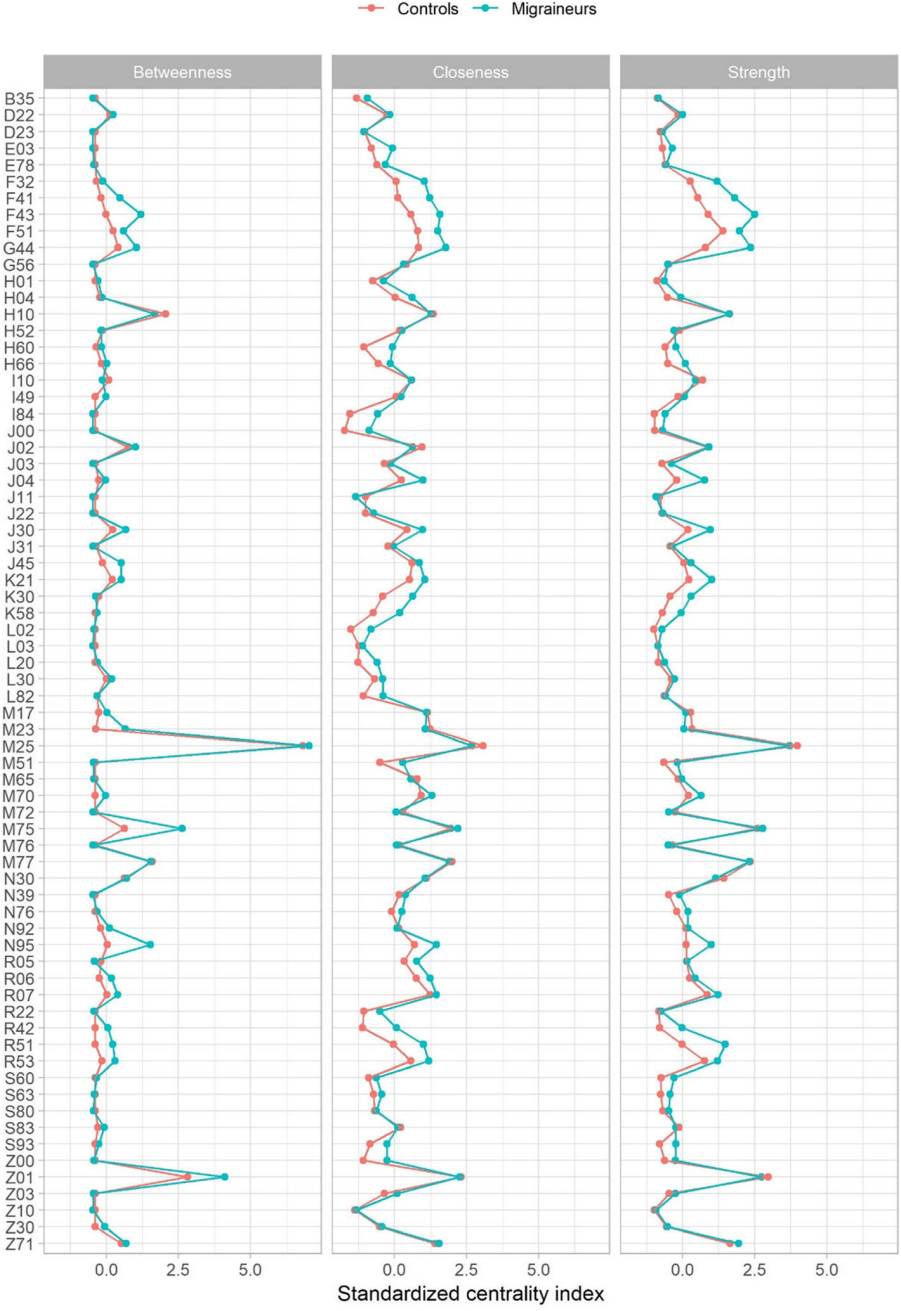
Three centrality measures visualized for migraineurs and controls. These have been calculated from the PDNs in Fig. 2. Betweenness: how well a comorbidity connects other comorbidities; Closeness: how close the comorbidity is to other comorbidities in the PDN; Strength: the sum of phi correlations over all connected comorbidities. Note that instead of the raw centrality measure values, the standardized values (i.e. Z-scores) have been plotted. This enables the comparison of the values from the two PDNs. Generally, the centrality of the comorbidites in migraineurs follows the same pattern as in controls, however, there appears to be some differences in betweenness in F-codes, M75, N95 & R-codes

Multiple descriptive network measures were calculated from the PDNs for migraine and controls. See Appendix 1 for a summary on the calculation and interpretation of these measures. The modularities related to the clustering were 0.23 and 0.31 for the migraine and controls, respectively, indicating that the obtained clusterings were sensible. The difference in modularity values likely reflects the increased overall morbidity in migraine patients leading to higher phi-correlations and greater overall connectivity in the PDN. Regressing the degrees pertaining to each comorbidity in migraine on the controls shows that the degree in controls predicts the degree in migraine patients well; on average one significant phi-correlation in controls implies 1.4 significant phi-correlations in migraine patients per comorbidity (regression slope 1.4, *p* < 0.001, Fig. 3). However, three outliers to this are detected using the mean-shift outlier test, namely F43 (reaction to severe stress, and adjustment disorders), G44 (other headache syndromes), and R51 (headache) (Bonferroni corrected *p*-values <0.001 for all three diagnosis codes). Across the centrality measures comorbidities in migraine patients show increased connectivity indicating that a patient is more likely to be affected by multiple conditions (Fig. 4). The betweenness measure in Fig. 4 shows clearest differences between migraine and controls at F-codes, M75 (shoulder lesions) and N95 (menopausal and other perimenopausal disorders) and R-codes.

**Fig. 5:**
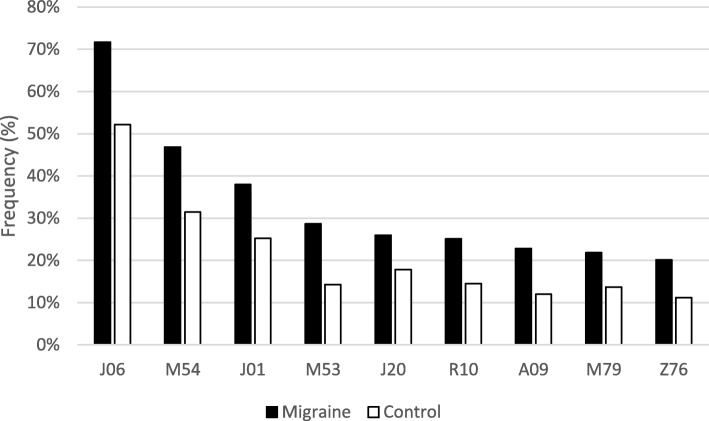
The frequency of diagnoses with > 20% prevalence in the migraine patients. J06 Acute upper respiratory infections of multiple and unspecified sites, M54Dorsalgia, J01 Acute sinusitis, M53 Other dorsopathies, J20 Acute bronchitis, R10 Abdominal and pelvic pain, A09 Diarrhoea and gastroenteritis of presumed infectious origin, M79 Other soft tissue disorders, Z76 Persons encountering health services in other circumstances

The 101 diagnostic codes that were present with a frequency of 2.5%–20% in migraine patients and had at least 2 significant phi-correlations included in the PDNs, exhibited significantly higher prevalence among migraine patients when compared to controls (Table 2). Figure 5 represents diagnoses with over 20% prevalence in migraine patients that were also significantly more frequent when compared to controls. The diagnostic code Z76 includes a group of heterogeneous sub-diagnostic codes related to health services and consisted mainly of the code Z76 as well as Z76.0 indicating issues of repeated prescriptions.

**Fig. 6:**
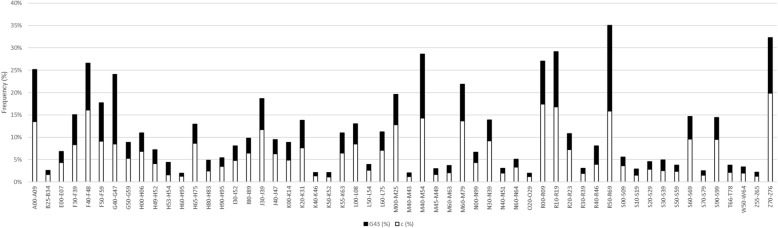
Overall prevalence (%) of diagnoses with > 2% prevalence in migraine patients with over 1.5-fold increase in migraine patients (black) compared to controls (white), *p* < 0.001 for all ICD-10 blocks. Bars are superimposed on each other. A00-A09 Intestinal infectious diseases, B25-B34 Other viral diseases, E00-E07 Disorders of thyroid gland, F30-F39 Mood [affective] disorders, F40-F48 Neurotic, stress-related and somatoform disorders, F50-F59 Behavioural syndromes associated with physiological disturbances and physical factors, G40-G47 Episodic and paroxysmal disorders, G50-G59 Nerve, nerve root and plexus disorders, H00-H06 Disorders of eyelid, lacrimal system and orbit, H49-H52 Disorders of ocular muscles, binocular movement, accommodation and refraction, H53-H54 Visual disturbances and blindness, H60-H95 Diseases of the ear and mastoid process, H65-H75 Diseases of middle ear and mastoid, H80-H83 Diseases of inner ear, H90-H95 Other disorders of ear, I30-I52 Other forms of heart disease, I80-I89 Diseases of veins, lymphatic vessels and lymph nodes, not elsewhere classified, J30-J39 Other diseases of upper respiratory tract, J40-J47 Chronic lower respiratory diseases, K00-K14 Diseases of oral cavity, salivary glands and jaws, K20-K31 Diseases of oesophagus, stomach and duodenum, K40-K46 Hernia, K55-K63 Other diseases of intestines, L00-L08 Infections of the skin and subcutaneous tissue, K50-K52 Noninfective enteritis and colitis, L50-L54 Urticaria and erythema, L60-L75 Disorders of skin appendages, M00-M25 Arthropathies, M40-M43 Deforming dorsopathies, M40-M54 Dorsopathies, M45-M49 Spondylopathies, M60-M63 Disorders of muscles, M60-M79 Soft tissue disorders, N00-N99 Diseases of the genitourinary system, N30-N39 Other diseases of urinary system, N40-N51 Diseases of male genital organs, N60-N64 Disorders of breast, R00-R09 Symptoms and signs involving the circulatory and respiratory systems, O20-O29 Other maternal disorders predominantly related to pregnancy, R10-R19 Symptoms and signs involving the digestive system and abdomen, R20-R23 Symptoms and signs involving the skin and subcutaneous tissue, R30-R39 Symptoms and signs involving the urinary system, R40-R46 Symptoms and signs involving cognition, perception, emotional state and behaviour, R50-R69 General symptoms and signs, S00-S09 Injuries to the head, S10-S19 Injuries to the neck, S20-S29 Injuries to the thorax, S30-S39 Injuries to the abdomen, lower back, lumbar spine and pelvis, S50-S59 Injuries to the elbow and forearm, S60-S69 Injuries to the wrist and hand, S70-S79 Injuries to the hip and thigh, S90-S99 Injuries to the ankle and foot, T66-T78 Other and unspecified effects of external causes, W50-W64 Exposure to animate mechanical forces, Z55-Z65 Persons with potential health hazards related to socioeconomic and psychosocial circumstances, Z70-Z76 Persons encountering health services in other circumstances

In addition, in order to get a more holistic view into multimorbidity, we also examined diagnoses on the block level. The whole spectrum of phenotypic diseasomes represented as ICD-10 blocks in migraine patients when compared to controls is visualized in Appendix 2. Migraine patients had an increase in overall diagnoses that were distributed across multiple ICD-10 code blocks. In Fig. 6, the blocks with a > 2% prevalence in the migraine cohort and an > 1.5-fold increase in prevalence compared to controls are presented. The largest enrichment was seen in visual disturbances, followed by episodic paroxysmal symptoms (G43* migraine code excluded). All together 56 blocks were enriched in patients with migraine (Fig. 6).

## Discussion

With this study, we investigated the phenotypic diseasomes associated with migraine in an occupational healthcare setting using PDNs and frequencies of ICD-10 codes when compared to age- and gender-matched control population. The most important results of the current study include 1) demonstrating that large datasets collected as a part of routine clinical praxis can be useful in naturally clustering diseasomes in an untargeted fashion; 2) diagnostic codes clustered differently into 9 and 6 clusters for migraine patients and controls, respectively; 3) the migraine PDN was larger and denser and exhibited one large cluster with functional-disorder-like symptoms including fatigue, respiratory, sympathetic nervous system, gastrointestinal, infection, mental and mood disorder diagnoses; 4) elucidating holistic and substantial multimorbidity for migraine seen as a holistic increase in prevalences of diagnoses across the whole ICD-10 coded diagnostic spectrum.

We have previously shown in the same population a substantial increase in healthcare visits and sick leaves for migraine patients compared to controls, and that less than 10% of these were linked to migraine in EMRs [26]. We further show here that this increase cannot be referred to a few driving morbidities but is associated with a generalized increase in multimorbidity. According to our best knowledge, this is the first untargeted approach to investigate the whole ICD-10 coded diseasome and PDN in migraine patients. Moreover, to our knowledge, this is the first study to include a matched control population for comparison when constructing PDNs. No other study has before elucidated this holistic and substantial multimorbidity for migraine. It is hoped that this study is followed by many more resulting in better understanding causes and consequences of migraine multimorbidity.

### Increased comorbidity based on phenotypic disease networks

Not only were there more clusters forming for the migraine group, but also the clusters included different diagnostic groups than for controls. There were two larger clusters forming with multiple diagnostic classes (M1 and M2 in migraine as well as C1 and C4 for controls). It was challenging to find a single nominator for these groups and thus clusters have been named by numbers and indicated by colours.

Interestingly, in migraine, the largest cluster M1, consisted of many co-existing morbidities already suggested to be related to migraine. These disorders quite well mimic those also found in functional disorders, and this cluster could potentially be called “a functional disorder-like”. As functional disorders have unknown causes but similar symptomology, it may be of interest to examine more underlying migraine in functional disorders or understand better the generalized symptoms apparently related to migraine [29]. Sympathetic disorders in cluster M1 and nerve root disorders in M2 represent an interesting common finding linking both clusters into the nervous system.

Increased connectivity in the PDN for migraine patients translates to higher morbidity meaning that a migraine patient is more likely to be affected by multiple conditions compared to controls. This was seen in the larger and denser PDN and the increased overall connectivity in all four centrality measures as well as 56 blocks shown in Fig. 6 exhibiting 1.5-fold increase in migraine. Still, the betweenness measure hinted at some diagnoses being more central in migraineurs than in controls (F-codes, shoulder lesions, menopausal and other perimenopausal disorders, R-codes), which may indicate that these comorbidities are more important for the emergence of morbidity in migraine patients than in controls. It remains to be seen whether targeting some of these conditions may be beneficial in minimizing the multimorbidity among migraine patients.

In addition, outliers were detected in regressing the number of significant phi-correlations in migraineurs on controls, namely reaction to severe stress (F43), other headache syndromes (G44), and headache (R51). Whether some of the controls influencing the formation of cluster C4 (Fig. 2) represent undiagnosed migraine cases, needs further research. It may be of interest removing controls with R51 diagnosis as potential migraine to further evaluate whether and how this would influence clustering in non-migraine population.

Some more detailed diagnosis differences were evident in migraine patients. Unlike in controls, fatigue (R53) and healthcare visits regarding problems related to life management difficulty (Z73) correlated in the cluster M1 in migraine patients. The latter did not exhibit significant phi-correlations in controls and is thus missing from PDNs. The prevalence was over two-fold among migraine compared to controls although causes for this cannot be determined in this study.

Non-headache symptoms and pain are common in migraine although pathophysiological causes may be complex and are not well understood [30, 31]. This was also seen in our study as frequencies of diagnoses for injuries, musculosceletal, connective tissue and nerve root disorders followed the same general pattern of increase in migraine despite clustering in the same fashion for both migraine patients and controls. Moreover, over 1.5-fold increase was detected for vestibular function, visual disturbances and dizziness (H81, H53, R42). It is possible that these changes may reflect migraine comorbidity related to balance and postural impairment as previously shown [14, 32–34]. Moreover, these may be linked to challenges in bodyboard control as detected in clinical practice for migraine patients.

Some diagnoses were in PDNs for migraine patients that were lacking in control networks; sleep disorders (G47) in cluster M1, acne (L70) and rosacea (L71) in cluster M7, and urticaria (L50) in cluster M6. The connection between migraine and sleep disorders has been recognized [35, 36]. However, the connection of migraine and rosacea and other skin disorders is only beginning to emerge, and studies have mainly focused on the increase in odds ratio of migraine in patients with rosacea, not the other way around [37, 38]. There are potential pathophysiological overlaps with migraine and neuroendocrine-immune-related skin disorders, in which also calcitonin gene related peptide (CGRP) has been suggested to play a role. Moreover, the role of vascular changes in rosacea and migraine are not fully understood but remain an interesting hypothesis also when reflecting on genetic studies [21, 38, 39].

### Increased comorbidity based on diagnostic codes and blocks

The study provided new insight to the migraine related diseasome, and we detected a global holistic increase in frequencies in more abundant diagnostic codes or blocks in migraine patients when compared to controls. The results support previous finding on migraine comorbidity with gastrointestinal, endocrinological, musculosceletal, neurological, and psychiatric disorders as well as with asthma and allergies [9–17, 19, 30]. Moreover, surprisingly many diagnostic codes related to skin, visual and hearing disorders were identified to be more common in migraine. Cardiovascular disorders played a less significant role in the PDNs and only few diagnoses were significantly increased among migraine patients when compared to controls. The reason for the latter may be that the occupational healthcare registry studied here mainly involves outpatient care, and cardiovascular complications are often treated at inpatient care. Increased morbidity of all therapy areas seems to correlate with a recent study by Ziegeler et al. (2019), where they examined all specialists which were consulted due to migraine in an outpatient setting, cardiologists had not been consulted here either, probably due to similar reasons [40].

In addition, several symptoms such as nausea and vomiting, dizziness, malaise and fatigue, and dyspepsia, just to mention a few, were more abundant in migraine when compared to controls. We have previously shown that regardless of increased healthcare resource utilization and prescribed sick-leave days, only less than 10% were directly linked to migraine diagnoses G43*. Our findings of increased co-existing morbidities including potential migraine-related symptoms highlight the difficulty in elucidating the true disease burden directly linked to migraine as it is difficult to determine which diseases and conditions should be included. Regardless, the results of this study provide new insight on a significant and surprisingly holistic multimorbidity related to migraine. Whether this is due to a general lower threshold responding to different stimuli that might make migraine patients seek healthcare more often than those without migraine, or due to some other pathophysiological reasons causing increased generalized sensitivity to comorbidities, needs further investigation.

### Limitations and strengths

There are some limitations typically associated with retrospective database analyses, as well as those associated with the cohort selection, many of these have been previously discussed [26]. In addition, real world variation in clinical praxis when examining ICD-10 coded diseasomes cannot be ruled out as subjects may have seen multiple healthcare providers with a variety of specialties. There is thus a risk that some information may not have been consistently recorded for all patients, potentially impacting the population sizes and other outcomes. For example, as we have examined a registry collected as a part of routine clinical practice in an outpatient occupational healthcare setting, some subjects may have additionally visited other healthcare instances e.g. public hospitals for emergency room visits or longer-term specialized healthcare. Another limitation is that as migraine is well known to be an underdiagnosed disease [26, 41, 42], it is possible that undiagnosed migraine patients in the control group may have confounded the study outcomes.

Notably, the study does not differentiate on disease severity as headache diaries are not yet included in EMRs in the registry. The study is thus based solely on diagnostic codes detected in EMRs, and ICD-10 codes do not separate between episodic and chronic migraine. We have previously reflected on a population receiving and failing prophylaxis for migraine, as having more severe migraine [26]. However, it was not reasonable to include these as a separate group for the diseasome analysis as they only represent 13% of the examined migraine sample.

Yet, from whatever aspect we chose to examine the morbidity for migraine, the multimorbidity was substantially increased when compared to age- and sex-matched controls without migraine diagnosis. This study, and others, raises questions on comorbidity definition. Is it an increase in frequency of individual diagnoses or should it be based on differences in diagnostic patterns compared to the general population, or as in e.g. genomics, defined by comparing study populations to larger general population samples [43]. Or should more specific methodologies be used to understand disease patterns? In this study we have deliberately taken the decision to discuss about co-existing morbidities and multimorbidity. It is hoped that further methodological and scientific development will take place in evaluating and defining what is really considered as comorbidity.

The strengths of this study partially reflect the limitations, and some of these have been discussed previously [26]. Notably, we have here examined migraine patients truly in a real world setting, and elucidated morbidity of individuals detected as a part of routine clinical praxis reflecting the outcomes resulting from a complex pathophysiological and socio-cultural environment [23]. In addition to previous studies, it is evident that there is substantial multimorbidity in migraine. It may also be important to examine risk patterns of migraine in a time-dependent manner. This especially, when morbidities may play a role in migraine chronification [44]. We evaluated the association between comorbidities in PDNs, but not the causality since it would have required directional networks and assessing comorbidities with respect to their timing [25]. Moreover, it is evident that the findings may reflect potentially migraine pathophysiology, drug-related adverse events, and even central sensitisation known to complicate the disease symptomology.

### Conclusions

We have here examined in a holistic and untargeted fashion migraine morbidity. Our data support the previous findings on many of the co-existing morbidities and potential comorbidities for migraine but also brings new insight on the vastness of the morbidity pattern. Regardless of the methodological approach, we detected a holistic increase in multimorbidity among migraine patients when compared to controls across the whole overall ICD-10 coded phenotypic diseasome. Our findings clearly reflect how migraine is observed in a socio-cultural environment and interpreted as a part of routine clinical praxis. As the first untargeted approach to elucidate migraine morbidity, our study may pose a benchmark and a baseline in understanding migraine morbidity detected as ICD-10 codes in the clinical praxis spectrum. More studies are warranted in understanding the pathophysiological causes and consequences for the findings. New migraine treatments may offer a solution in understanding migraine pathophysiology and morbidity patterns through effectiveness analysis focused on the holistic disease burden outcomes in real world settings.

## Figures and Tables

**Fig. 7:**
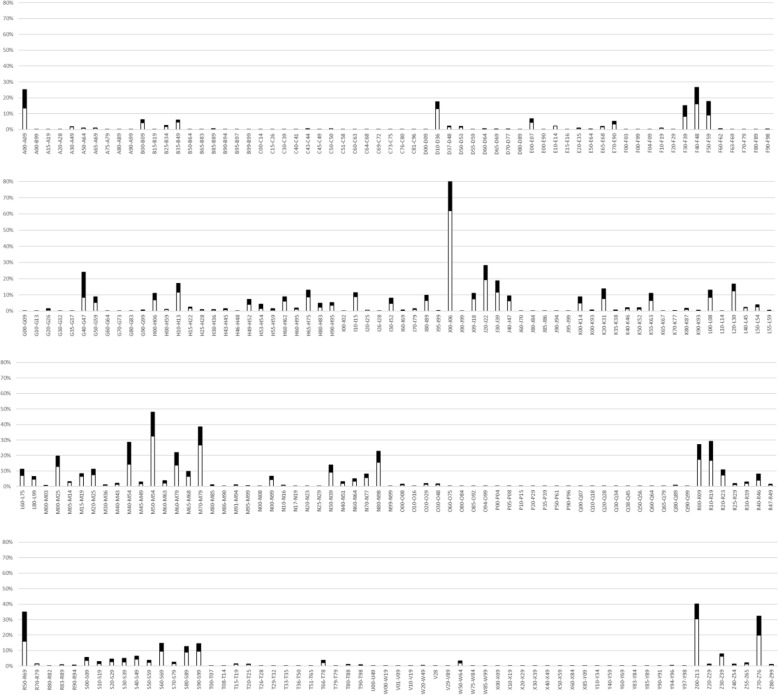
Prevalence (%) of all ICD-10 blocks in patients with migraine (black) and controls (white). Bars are superimposed on top of each other

## Data Availability

Suomen Terveystalo Plc. authorities are responsible for administrative decisions controlling access to EMR data and ensuring data privacy according the Finnish laws.

## Appendix 1

**Table 3:** Summary of the four reported connectivity measures from the PDNs. Degree, betweenness, closeness, and strength are defined for single diagnosis codes, while modularity is defined for the whole PDN based on a given clustering. For mathematical and formal definitions see [28] and [26]. Note that the C in the value range column stands for the total number of all diagnosis codes in the PDN

Network measure	Calculation	Value range	Interpretation
Degree	Number of phi correlations shared with other diagnosis codes.	Min: 2 (or 0) Max: C – 1	Simplest centrality measure that gives a general idea how involved a diagnosis code is with other diagnosis codes. Only the directly connected diagnosis codes are accounted for. *For the analyses in this study, all diagnosis codes with at most one significant phi-correlation were excluded, thus the minimum degree in PDN is 2, and not 0.
Strength	Sum of the phi correlations shared with other diagnosis codes.	Min: 0 Max: C - 1	Similar to degree but weighted with the phi correlations. A diagnosis code with a few very strong phi correlations and a diagnosis code with many weak phi correlations can have the same strength. Minimum value = There are no significant phi correlations. Maximum value = perfect phi correlation with all other diagnosis codes.
Betweenness	The number of shortest paths between any two diagnosis codes of which this diagnosis code is a part of divided by all possible paths. The paths are weighted by the phi correlations.	Min: 0 Max: 1	Most diagnosis codes have betweenness 0, since most diagnosis codes are not part of any shortest path [28]. This measure indicates how central one diagnosis code is relative to all other diagnosis codes. High betweenness may indicate that the diagnosis code mediates the correlation between other diagnosis codes, leads to many other diagnosis codes, or is the end point of many comorbidities.
Closeness	The inverse of the total sum of the lengths of the shortest paths to all other diagnosis codes. The path lengths are weighted by the phi correlations.	Min: 0 Max: 1	The less intermediary diagnosis codes there are between two diagnosis codes and the higher the phi correlations, the ‘closer’ the two diagnosis codes are. Closeness gives a measure of this for one diagnosis code averaged over all other diagnosis codes. High closeness of a given diagnosis code indicates that it is more central relative to the other diagnosis codes and may represent a clinically important aspect of the diseasome. Minimum value = no shortest paths lead through the diagnosis code. Maximum value = all shortest paths go through the diagnosis code and all shortest paths have length 1.
Modularity	Number of phi correlations within a cluster divided by the number of all phi correlations minus the expected proportion of phi correlations within a random cluster.	Min: − 1 Max: 1	Modularity can be used to compare different clustering methods or clustering of two networks with the same algorithm. Here used to sanity check the clustering from the Walktrap-algorithm. Modularity < 0: the clustering divides the PDN into subnetworks worse than expected by a random clustering. Modularity > 0: the clustering divides the PDN into subnetworks better than expected by a random clustering.

## Abbreviations

EMR: Electronic medical records
PDN: Phenotypic disease networks
